# Seasonal variation of BMI at admission in German adolescents with anorexia nervosa

**DOI:** 10.1371/journal.pone.0203844

**Published:** 2018-09-11

**Authors:** David R. Kolar, Katharina Bühren, Beate Herpertz-Dahlmann, Katja Becker, Karin Egberts, Stefan Ehrlich, Christian Fleischhaker, Alexander von Gontard, Freia Hahn, Michael Huss, Charlotte Jaite, Michael Kaess, Tanja Legenbauer, Tobias J. Renner, Veit Roessner, Ulrike Schulze, Judith Sinzig, Ida Wessing, Johannes Hebebrand, Manuel Föcker, Ekkehart Jenetzky

**Affiliations:** 1 Department of Child and Adolescent Psychiatry, University Medicine of the Johannes Gutenberg-University, Mainz, Germany; 2 Department of Child and Adolescent Psychiatry, Psychosomatics and Psychotherapy, University Hospital of the RWTH Aachen, Aachen, Germany; 3 Department of Child and Adolescent Psychiatry, Psychosomatics and Psychotherapy, Philipps-University Marburg and University Hospital Marburg, Marburg, Germany; 4 Department of Child and Adolescent Psychiatry, Psychosomatics and Psychotherapy, University Hospital Wuerzburg, Wuerzburg, Germany; 5 Eating Disorders Research and Treatment Center, Department of Child and Adolescent Psychiatry, Faculty of Medicine, TU Dresden, Dresden, Germany; 6 Division of Psychological & Social Medicine and Developmental Neurosciences, Faculty of Medicine, TU Dresden, Dresden, Germany; 7 Department of Child and Adolescent Psychiatry and Psychotherapy, University Medical Center Freiburg, Freiburg, Germany; 8 Department of Child and Adolescent Psychiatry Saarland University Hospital, Homburg, Germany; 9 Department of Child and Adolescent Psychiatry, Psychosomatics and Psychotherapy, LVR–Hospital Viersen, Viersen, Germany; 10 Department of Child and Adolescent Psychiatry, Psychosomatic Medicine and Psychotherapy, Charité –Universitätsmedizin Berlin, corporate member of Freie Universität Berlin, Humboldt-Universität zu Berlin, and Berlin Institute of Health, Berlin, Germany; 11 Clinic of Child and Adolescent Psychiatry, Center for Psychosocial Medicine, University Hospital Heidelberg, Heidelberg, Germany; 12 University Hospital of Child and Adolescent Psychiatry and Psychotherapy, University of Bern, Bern, Switzerland; 13 LWL University Hospital Hamm for Child and Adolescent Psychiatry, Ruhr University Bochum, Bochum, Germany; 14 Department of Child and Adolescent Psychiatry, Psychosomatics and Psychotherapy, University Hospital Tuebingen, Tuebingen, Germany; 15 Department of Child and Adolescent Psychiatry, Faculty of Medicine, TU Dresden, Dresden, Germany; 16 Department of Child and Adolescent Psychiatry/Psychotherapy, University Hospital, University of Ulm, Ulm, Germany; 17 Department of Child and Adolescent Psychiatry, Psychosomatics and Psychotherapy, LVR-Klinik Bonn, Bonn, Germany; 18 Department for Child and Adolescent Psychiatry, University Hospital Muenster, Muenster, Germany; 19 Department of Child and Adolescent Psychiatry, Psychotherapy, and Psychosomatics, University Hospital Essen, University of Duisburg-Essen, Essen, Germany; Charité-Universitätsmedizin Berlin, Campus Benjamin Franklin, GERMANY

## Abstract

**Objective:**

Recent preliminary studies indicated a seasonal association of BMI at admission to inpatient treatment for anorexia nervosa (AN), indicating lower BMI in the cold season for restrictive AN. An impaired thermoregulation was proposed as the causal factor, based on findings in animal models of AN. However, findings regarding seasonality of BMI and physical activity levels in the general population indicate lower BMI and higher physical activity in summer than in winter. Therefore, we aimed to thoroughly replicate the findings regarding seasonality of BMI at admission in patients with AN in this study.

**Method:**

AN subtype, age- and gender-standardized BMI scores (BMI-SDS) at admission, mean daily sunshine duration and ambient temperature at the residency of 304 adolescent inpatients with AN of the multi-center German AN registry were analyzed.

**Results:**

A main effect of DSM-5 AN subtype was found (*F*(2,298) = 6.630, *p* = .002), indicating differences in BMI-SDS at admission between restrictive, binge/purge and subclinical AN. No main effect of season on BMI-SDS at admission was found (*F*(1,298) = 4.723, *p* = .025), but an interaction effect of DSM-5 subtype and season was obtained (*F*(2,298) = 6.625, *p* = .001). Post-hoc group analyses revealed a lower BMI-SDS in the warm season for restrictive AN with a non-significant small effect size (*t*(203.16) = 2.140, *p* = .033; *Hedges*′*g* = 0.28). Small correlations of mean ambient temperature (*r* = −.16) and daily sunshine duration (*r* = −.22) with BMI-SDS in restrictive AN were found. However, the data were widely scattered.

**Conclusions:**

Our findings are contrary to previous studies and question the thermoregulatory hypothesis, indicating that seasonality in AN is more complex and might be subject to other biological or psychological factors, for example physical activity or body dissatisfaction. Our results indicate only a small clinical relevance of seasonal associations of BMI-SDS merely at admission. Longitudinal studies investigating within-subject seasonal changes might be more promising to assess seasonality in AN and of higher clinical relevance.

## Introduction

The current experience of eating disorder symptoms is influenced by the change of seasons [1–4], with a stronger seasonal association of symptom severity and mood in bulimia nervosa than in anorexia nervosa (AN) [2, 5–7]. Recent preliminary studies suggest a small to medium seasonal association with body mass index (BMI; kg/m^2^) in patients with AN admitted to inpatient treatment [8, 9], indicating that patients who were admitted in the cold season of the year showed a lower BMI at admission compared to patients admitted in the warm season. This difference was found only for patients with AN restricting subtype (ANR), whereas no difference was observed in patients with AN binge-eating/purging subtype (ANBP). A similar effect was observed in a small outpatient sample in the Netherlands [10]. In addition, a medium correlation between BMI at admission and the ambient temperature in the month of admission was found in patients with ANR but not in patients with ANBP [8]. This indicates a lower BMI of patients with ANR during cold ambient temperatures.

Fraga et al. [8] and Carrera et al. [10] conclude that lower body weight in winter in ANR is caused by a process of thermoregulation. They assume that less body insulation due to less subcutaneous fat in patients with ANR leads to a higher calorie consumption for warming up, resulting in a subsequently lower BMI during the cold season. Their conclusion is based on findings of an animal model of activity-based AN, where access to a warm plate and increased ambient temperature resulted in lower body weight loss or better weight restoration [11, 12]. However, it is questionable if these findings can be translated directly to humans, as humans can voluntarily compensate a lack of body insulation by clothing or heating in closed rooms. In addition, a thermoregulatory effect should also be visible in healthy populations, albeit to a lesser extent. However, the opposite was found, as higher BMI values were observed in the cold than in the warm season in general population studies [13–15]. Moreover, an increase in body weight in the cold season in adult populations [16] and a seasonal weight loss from spring to the beginning of winter in children and adults [17, 18] were observed.

In addition, several other potential explanations for a seasonal variation of BMI at admission besides thermoregulation were not considered by the previous literature. For example, less exposure to sunshine is associated with low vitamin-D serum concentrations, which are furthermore linked to an increase in depressiveness [19] and more emotional and peer problems in adolescents in general [20], although the findings are inconsistent in patients with AN [21, 22]. Depressiveness might further exacerbate body weight loss and result in a lower BMI. Furthermore, the promotion of restraint around New Year and dieting in summer months in women’s magazines as found in a content analysis [23], combined with more body exposure and comparison, for example in swimming pools or through more revealing summer clothing during warmer ambient temperatures, might further influence weight loss in AN. The latter might even indicate a lower BMI at admission in summer months in opposite to the findings of the previous studies.

In addition, the previous studies on the seasonal association of BMI of patients with AN at admission showed several methodological limitations. First, only absolute BMI values were compared, although two of the studies investigated adolescents [8, 10]. Absolute BMI values are not recommended to measure underweight in adolescence, as development-related variations might bias results in this age group [24]. Hence, the significance of the difference is difficult to determine, and age- and gender-standardized BMI scores (BMI-SDS) might represent a more accurate measure. Second, weather data were collected at the clinical site and not at the residence of the patients, which might confound the association of ambient temperature and BMI at admission [8–10]. Third, only one of the studies directly focused on the association of season with BMI at admission [8] and none of the researchers corrected their findings for multiple testing, increasing the risk of false positive findings [25]. Finally, one study was conducted in a subsample of patients admitted to a specialized refeeding unit with severe and enduring AN and a mean BMI below 13 [9]. The latter findings might not be generalizable to all patients with AN, especially adolescent patients, who often show a shorter duration of illness at admission compared to adults with severe and enduring AN.

In view of the presented concerns regarding the previous studies on seasonal associations of BMI in patients with AN admitted to inpatient treatment, we aimed to replicate these findings in a rigorous analysis of a nationwide sample of adolescents with AN from the multicenter German registry for AN [26]. In particular, we aimed to assess whether BMI at admission truly is lower in the cold than in the warm season in adolescents with AN and whether this association is dependent on the subtype of AN. We expected lower BMI-SDS values in the cold compared to the warm season for ANR, and no seasonal differences for ANBP and subclinical AN. In contrast to previous studies and to improve data quality, climate data were measured at the residence of the patients rather than at the location of the treatment center, covering latitudes ranging from approximately 54° 05’ to 47° 39’. Furthermore, we used BMI-SDS instead of absolute BMI, as the former is a more reliable measure for adolescents with AN. In addition, we investigated the association of BMI-SDS with relevant climate factors such as ambient temperature and mean sunshine duration in the time prior to hospitalization, expecting a positive correlation based on the findings of the previous studies.

## Methods

### Participants

In the present study, data of patients of the multi-center German registry of children and adolescents with AN (Kompetenznetz Anorexie-Register e.V.) were analyzed. Enrolled patients were admitted to inpatient treatment in 16 child and adolescent psychiatry centers between August 2014 and May 2017 (centers as reported in Bühren et al. [26], with the addition of two new centers in Mainz and Muenster, Germany). Written informed consent was obtained from all patients and their legal guardians. The study was approved by the ethics committees of all participating centers following the umbrella ethic votes by the initial centers Aachen and Essen (ethics committee of the medical faculty Aachen, Germany, ref. nr. EK028/12, and ethics committee of the medical faculty Duisburg-Essen, ref. nr. 12-5169-BO; for a full list of all ethics committees please refer to S1 File). Only complete records of patients consisting of date of admission, age, weight, height and diagnosis of DSM-5 AN subtype at the time of admission, and gender were included in the study. DSM-5 diagnoses were clinical diagnoses by the clinician in charge at the local center. DSM-5 criteria were presented within the registry entry form with a checklist to increase reliability of the diagnoses. Subclinical AN was defined as cases of AN in which not all criteria for AN were completely fulfilled, but the overall clinical impression was considered as AN. Due to economic reasons, no standardized diagnostic procedure (e.g. interview or questionnaire) was established for participating centers. BMI-SDS was calculated in reference to Kromeyer-Hauschild et al. [24].

### Climate data

**Fig 1:**
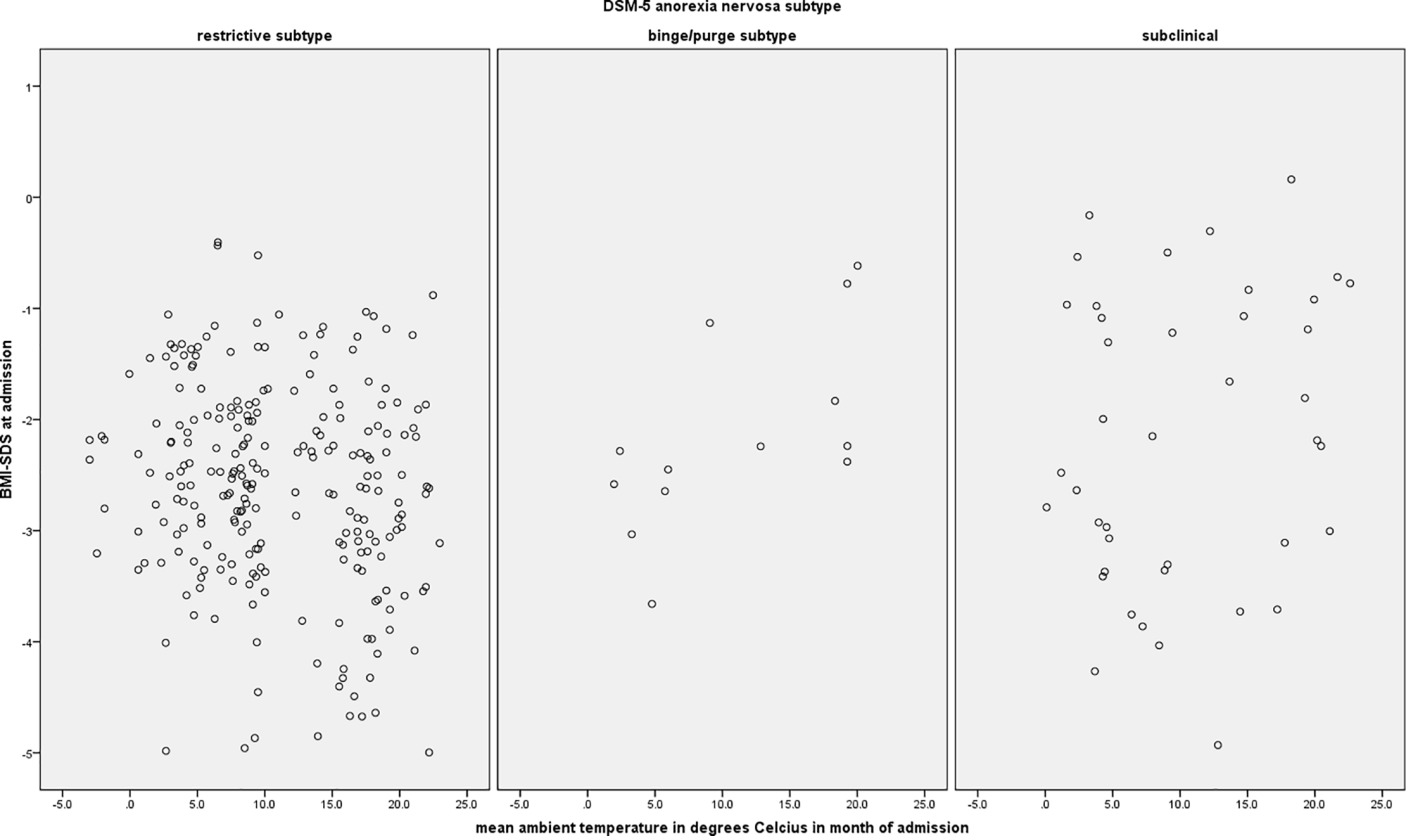
Scatterplot of the mean ambient temperature in the month of admission.

Climate data on daily mean temperature in degrees Celsius and sunshine duration in hours at the residence of the patients were obtained from the German Weather Service (DWD, Deutscher Wetterdienst) for all records with postal code of residence. As postal codes were reduced to the first two numbers due to anonymization, the official weather station of the DWD attributed to the postal region was used as the data source. In the case of two or three attributed stations, the mean value of the stations was used. For each participant, we calculated mean values of ambient temperature and sunshine duration for the month of admission, 30 days previous to the month of admission, and 31 to 60 days previous to the month of admission. Although four distinct seasons can be observed in Germany, the distribution of the temperature data (cf. Fig 1) does not clearly promote a four-seasonal structure but rather a warm/cold temperature division [27]. Hence, we considered the date of admission as lying in the cold season if the German mean temperature of the month was below the average in that separate year of admission according to the DWD, and in the warm season if it was above the yearly average of Germany, respectively. With the exception of 2014, the cold season lasted from October to April and the warm season from May to September. October 2014 was unusually warm, and therefore three cases with admission in this month were coded as belonging to the warm season.

### Statistical analyses

BMI-SDS as the dependent variable was tested for normal distribution with the Shapiro-Wilks test. The influence of both DSM-5 subtype of AN and the season on the BMI-SDS was analyzed using a two-way analysis of variance (ANOVA). DSM-5 subtype, season and interaction of subtype and season were analyzed as between factors. Subsequently, two one-way ANOVAs were conducted to assess DSM-5 subtype differences separately for warm and cold season. Unpaired t-tests were calculated to assess seasonal differences within the AN subtype samples for further exploration. The overall alpha-error level was set at α = .05. Bonferroni-corrected alpha-levels were *α* = .00625 for the eight tests conducted. Finally, Pearson correlations of ambient temperature, sunshine duration and BMI-SDS were calculated for each AN subtype separately on an exploratory level.

## Results

Of a total of 339 admissions recorded in the registry, 304 records reporting BMI-SDS at admission and DSM-5 subtype were obtained. Participants were aged 8.9 to 19.3 years at admission, and showed a BMI range of 11.39 to 20.69 kg/m^2^ (BMI-SDS: -5.00 to 0.16). Table 1 provides information on the distributions of gender, season of admission, first inpatient admission for AN treatment, age, BMI, and BMI-SDS separated for DSM-5 AN subtypes. BMI-SDS was normally distributed within the total sample (Shapiro-Wilks test, *W*(304) = .995, *p* = .520). Mean temperatures (in degrees Celsius) and mean daily sunshine durations (in hours) in the month of admission differed significantly between the cold and warm season (Temperature: cold season: *M* = 5.87, *SD* = 3.17; warm season: *M* = 17.52, *SD* = 2.76; *t*(284.84) = −33.66, *p* ≤ .001; Sunshine duration: cold season: *M* = 2.93, *SD* = 1.47; warm season: *M* = 6.77, *SD* = 1.49; *t*(265.61) = −21.98, *p* ≤ .001).).

**Table 1:** Distribution of DSM-5 subtypes, gender, age, BMI and BMI-SDS in the sample.

	DSM-5 anorexia nervosa subtype							
	Restricting type		Binge-eating/purging type		Subclinical		total	
	*N*	*%*	*N*	*%*	*N*	*%*	*N*	*%*
Total patients	249	81.9	13	4.3	42	13.8	304	100
*Gender*								
Male	7	2.8	0	0	3	7.1	10	3.3
Female	242	97.2	13	100	39	92.9	294	96.7
First inpatient admission	175	70.3	4	30.8	33	78.6	212	69.7
	*M*	*SD*	*M*	*SD*	*M*	*SD*	*M*	*SD*
Age (years)	15.02	1.78	16.97	1.20	14.77	1.96	15.07	1.82
BMI (kg/m^2^)	15.07	1.39	16.59	1.40	15.72	2.15	15.22	1.55
BMI-SDS	-2.62	0.92	-2.14	0.87	-2.20	1.31	-2.54	1.00
Duration of illness (months) ^†^	11.81	12.14	20.18	14.23	11.60	7.63	12.11	11.84
*Season*	*N*	*%*	*N*	*%*	*N*	*%*	*N*	*%*
Cold	145	81.5	7	3.9	26	14.6	178	58.6
Warm	104	82.5	6	4.8	16	12.7	126	41.4

^†^Due to missing values, duration of illness was only obtained from 282 Patients in total, which were 236 patients with restrictive type, 11 patients with binge-eating/purging type and 35 patients with subclinical anorexia nervosa.

**Fig 2:**
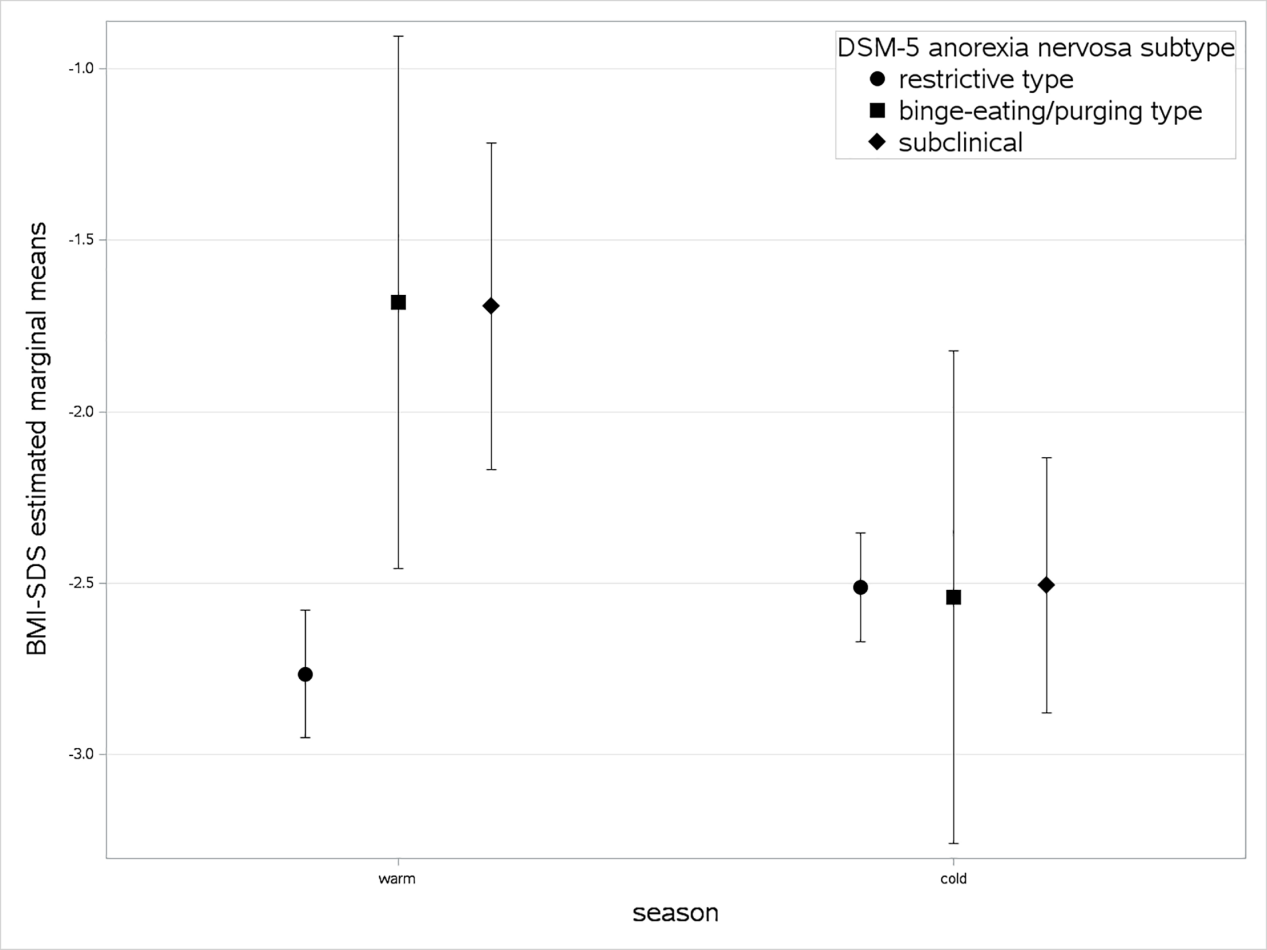
Estimated marginal means of the BMI-SDS for the DSM-5 anorexia subtypes. Note: Error bars are 95% confidence intervals.

A two-way ANOVA was conducted to examine the effects of DSM-5 subtype and season on the BMI-SDS. A main effect was found regarding DSM-5 subtype (*F*(2,298) = 6.630, *p* = .002), whereas no main effect of season was found (*F*(1,298) = 4.723, *p* = .025). A DSM-5 subtype × season interaction effect was found (*F*(2,298) = 6.625, *p* = .001). Fig 2 shows the interaction diagram of the DSM-5 subtypes of AN with season, presenting the marginal means and corresponding confidence intervals. The lowest BMI-SDS values of ANR were observed in the warm season, with a large difference of ANR from other AN subtypes (*F*(2,123) = 10.492, *p* ≤ .001), whereas in the cold season, AN subtypes did not vary regarding their BMI-SDS at admission (*F*(2,175) = 0.004, *p* = .996). To investigate possible seasonal differences within AN subtypes, post-hoc t-tests were conducted. Table 2 reports BMI-SDS means, standard deviations and t-test statistics separately for each DSM-5 subtype of AN. A significant difference was only found in the ANR group, indicating lower BMI-SDS in the warm than in the cold season (*t*(232.09) = 2.881, p = .004). According to Cohen[28], effect sizes of the overall DSM-5 subtype main effect and the overall DSM-5 subtype and season interaction effect were small (partial eta-squared of both .043). A medium effect size was found for DSM-5 subtype differences in the warm season ($\eta_{p}^{2}=.14$) and a small effect size was found for seasonal difference on BMI-SDS in the ANR group (*Hedges*′*g* = 0.28, 95% CI [0.02, 0.53]). Similar results were obtained when using BMI instead of BMI-SDS (see S1 File), although the effects failed to reach significance when applying Bonferroni-corrected alpha error levels.

**Table 2:** Mean and SD values of BMI-SDS and post-hoc t-tests separated for DSM-5 subtype and season.

DSM-5 anorexia nervosa subtype	Cold season			Warm season						
	*N*	*M*	*SD*	*N*	*M*	*SD*	*t*	*df*	*p*	*Hedges’ g (95% CI)*
Restricting type	145	-2.51	0.86	104	-2.76	0.97	2.14	203.16	.033	0.28 (0.02, 0.53)
Binge-eating purging type	7	-2.54	0.77	6	-1.68	0.79	-1.98	11	.072	-1.03 (-2.19, 0.13)
Subclinical	26	-2.51	1.30	16	-1.69	1.20	-1.43	40	.050	-0.63 (-1.27, 0.01)

**Fig 3:**
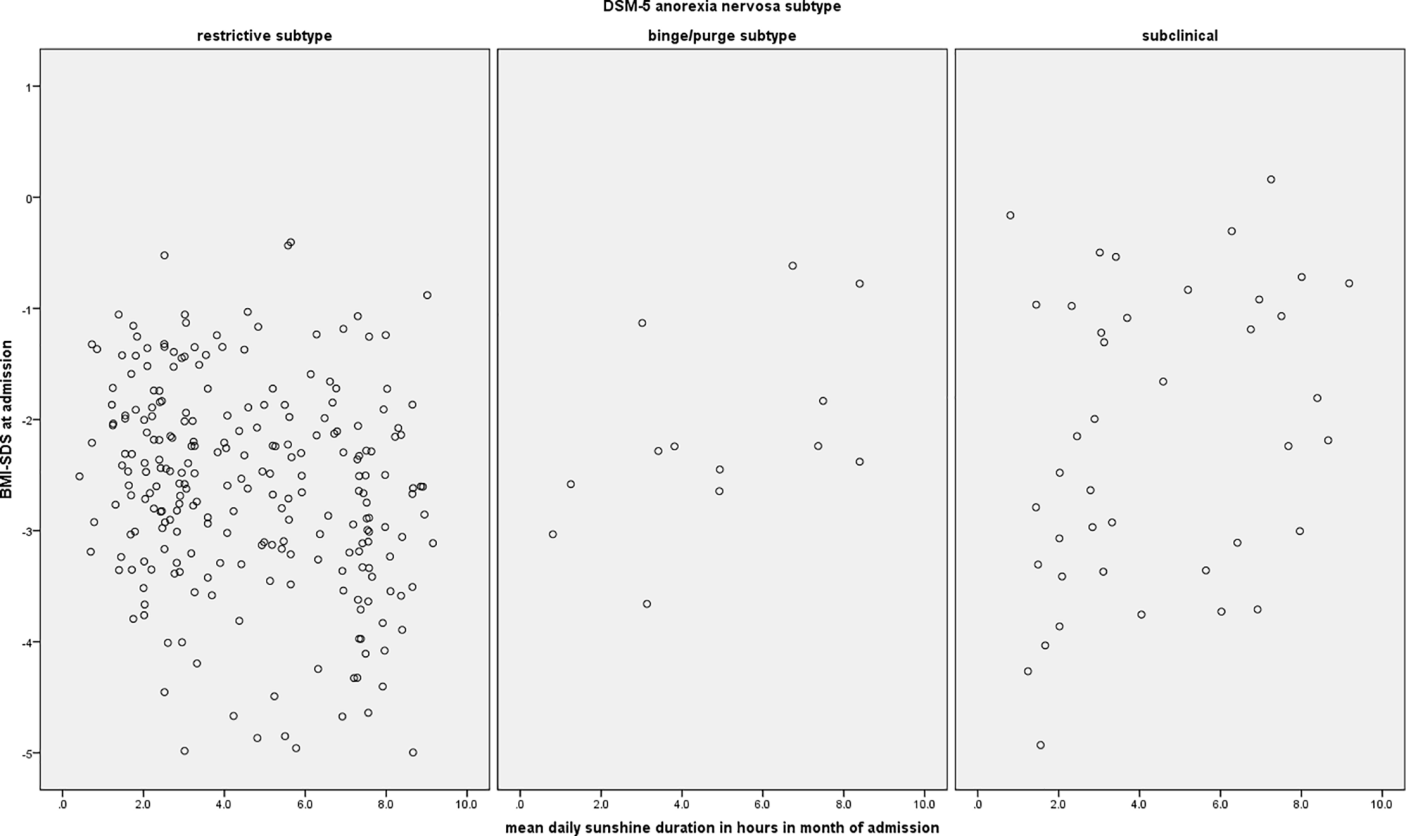
Scatterplot of the mean daily sunshine duration in the month of admission.

In Table 3, the correlations of BMI-SDS with ambient temperature and sunshine duration in the month of admission, 1 to 30, and 31 to 60 days prior to admission are shown separately for each DSM-5 subtype of AN. Figs 1 and 3 show the data pattern of ambient temperature and sunshine duration in month of admission in relation to BMI-SDS at admission. Mean daily ambient temperature and sunshine duration showed a small negative correlation with BMI-SDS in the ANR group in the month of admission, and sunshine duration also in the month prior to admission. Significant correlations of ambient temperature and mean daily sunshine duration with BMI-SDS were also found in the ANBP group, whereas no significant correlations were found in the subclinical AN group. However, the sample size of the ANBP group was too small to derive meaningful conclusions.

**Table 3:** Correlations of daily ambient temperature (degrees Celsius) and daily sunshine duration in hours with BMI-SDS separated for DSM-5 anorexia nervosa subtypes.

		Mean daily ambient temperature (degrees Celsius)			Mean daily sunshine duration (hours)		
		Month of admission	-30 days	-31 to -60 days	Month of admission	-30 days	-31 to -60 days
DSM-5 subtype	*N*	*r* (*p*)	*r* (*p*)	*r* (*p*)	*r* (*p*)	*r* (*p*)	*r* (*p*)
Restricting type	242^†^	-.161 (.012*)	-.116 (.071)	.005 (.936)	-.218 (.001**)	-.171 (.007**)	-.115 (.074)
Binge-eating purging type	13	.626 (.022*)	.635 (.020*)	.674 (.012*)	.505 (.079)	.596 (.032*)	.681 (.010**)
Subclinical	41	.177 (.269)	.151 (.347)	.059 (.715)	.296 (.061)	.128 (.424)	.063 (.694)

Ambient temperature and sunshine duration could only be obtained for cases reporting postal codes.

^†^One patient partially excluded from analysis as weather data were not obtainable for month of admission.

* p ≤ .05

** p ≤ .01

## Discussion

This study investigated the seasonal influence on the BMI-SDS of adolescents with AN. No main effect of season on BMI-SDS in AN was found, whereas a DSM-5 subtype main effect and a season × DSM-5 subtype interaction effect were found. Post-hoc analyses revealed that DSM-5 subtypes varied with regard to their BMI-SDS at admission in the warm but not the cold season. Furthermore, the BMI-SDS of patients with ANR subtype was lower when admitted in the warm season compared to the cold season, although not statistically significant after Bonferroni-correction. No such difference was observed in subclinical AN or ANBP. This indicates that the interaction effect of DSM-5 subtype and season is mainly driven by the ANR subtype, although the ANBP sample size was too small to derive any meaningful conclusion for this subgroup. Furthermore, we found a small negative association of BMI-SDS with ambient temperature and daily sunshine duration, also indicating a lower BMI-SDS in summer than in winter months. However, considering the pattern of the scatterplots of mean ambient temperature and mean sunshine duration with BMI-SDS at admission, the correlations appear to be of only statistical significance and of little clinical relevance, as the data are widely scattered.

Fraga et al. [8] strongly advocate a thermoregulatory mechanism underlying the association of season with BMI in AN. However, our data do not support this hypothesis and indicate that seasonal variation of BMI in AN is most likely more complex. This is in line with Nielsen[29], who found, for example, two annual admission peaks for AN in winter and summer. Several other biological, behavioral, cognitive and even structural factors such as growth, changes in physical activity or body dissatisfaction, depressiveness, or waiting time, might confound an overly simplified association of season with BMI, as these factors themselves might be subject to seasonal variation. For example, physical activity in AN shows seasonal variation, although the literature is inconsistent if physical activity in AN is increased in summer or winter [10, 30]. It is also possible that the summer holidays could influence when AN patients are remitted to inpatient treatment. It should be furthermore noted that in our study, the association of a lower BMI with season is similar to observations in the general population [14, 15]. Thus, finding a similar pattern in patients with ANR might not be disorder-specific but rather reflect the general seasonality of body weight. In addition, the onset of AN is often associated with a prior period of dieting, and there is preliminary evidence that dieting behaviors increase in spring [31]. As patients with ANR generally need a higher calorie intake for weight maintenance compared to patients with subclinical AN or ANBP, seasonal effects might be more noticeable in ANR [32], and overall favor a lower BMI in summer than in winter. Differences in health care systems might also explain differences between preliminary studies and our data. In Germany, clear treatment guidelines exist, recommending early inpatient treatment for adolescents with AN [33]. Finally, the clinical relevance of the association of season with BMI at admission appears to be arguable, as effect sizes and correlations are rather small and preliminary studies translating the thermoregulatory hypothesis into a warming treatment for AN showed only mixed results regarding efficacy [34–37].

In addition, there are several methodological differences that might further explain the discrepant findings. None of the previous studies corrected their findings for multiple testing, although a variety of tests were conducted regarding seasonal associations. Compared to the studies by Fraga et al. [8] and Carrera et al. [10], our ANR sample was more than double the size and was comparable regarding age, BMI at admission and number of patients with previous episodes of inpatient treatment, which raises questions regarding the generalizability of their results given the medium effect sizes, small sample sizes and the large number of possible confounders. Furthermore, all hospital admissions of patients with AN were included in our study, whereas Fraga et al. [8] only considered patients who were admitted and discharged during the same season. This could have especially affected patients admitted in autumn and spring as well as severely ill patients, given that AN is a condition which often requires prolonged treatment. Furthermore, the previous studies used BMI instead of an age-adjusted measurement, even when investigating an adolescent population [8, 10], which might have confounded the data. However, in our study, we found the same direction of the association (lower BMI in warm season for ANR) regardless of whether BMI-SDS or BMI was applied.

Several strengths of our study have to be noted. Compared to previous studies on seasonality of BMI at admission in AN, this is the first multicenter study to result in a large sample of adolescents with AN. Collecting data from different hospitals increases the representativeness of the sample and decreases the influence of regional climate effects. In addition, the study included not only ANR and ANBP cases but also patients with subclinical AN, which constitute a significant proportion of all AN diagnoses in adolescence and should not be excluded a priori. However, no effects were found in the subclinical group in this population. Moreover, we assessed ambient temperature and sunshine duration at the residence of the patients rather than at the admission site in order to increase validity of the weather data. Lastly, this is the first study to report a correlation of sunshine duration with BMI-SDS in an AN sample.

Nevertheless, several limitations of our study also need to be considered. Perhaps the most important limitation is that due to German data safety laws, patients were not identifiable once entered into the register. Therefore, double entries might have occurred if a patient was admitted to two participating centers and did not report their previous participation. In addition, the date of admission was reduced to the month and the postal code from five to the first two digits prior to participation, which might have increased the error variance. The AN diagnoses in our study have to be considered as clinical diagnoses, as no standardized diagnostic procedure was applied across all centers. In addition, the prevalence of ANBP cases was lower than expected compared to other studies with adolescent AN samples. Given the high comorbidity of depression and AN, seasonal effects on depressiveness might moderate the BMI-SDS of individuals with AN. Unfortunately, depression as a diagnosis was not assessed systematically in the registry, which is a clear limitation. In addition, other behavioral factors related to season and BMI-SDS, such as physical activity, were not assessed in the registry either.

In conclusion, our study calls into question the assumed direction of a seasonal association of BMI at admission in AN as proposed in the previous literature and further questions the clinical relevance of seasonal differences in BMI merely at admission in cross-sectional studies. Future studies investigating seasonality in AN should consider emerging hypotheses such as for example possible effects of seasonality of poly-unsaturated fatty acids in AN [38]. Longitudinal studies examining the within-change of BMI over all seasons in individuals with AN might further enlighten the association of seasonality and BMI and be of greater clinical relevance, as confounding seasonal variables such as changes in body dissatisfaction, an increase in outdoor physical activity or changes in nutritional intake can be investigated directly and conclusions for treatment can be drawn.

## Supporting information

S1 File BMI analysis. Results of the main analysis by using BMI instead of BMI-SDS.
(PDF)

S2 File Ethics statement. List of all involved ethics committees.
(PDF)
