# Supplementary material for: Seasonal variation of BMI at admission in German adolescents with anorexia nervosa
Source: PLoS One. 2018 Sep 11;13(9):e0203844. doi: 10.1371/journal.pone.0203844 (PMC6133390; doi:10.1371/journal.pone.0203844)
Supplement: S1 File — Results of the main analysis by using BMI instead of BMI-SDS. (PDF) [file pone.0203844.s001.pdf]

Supplement 1 – Analysis of BMI instead of BMI-SDS

1. Two-way ANOVA to examine effects of DSM-5 subtype and season on BMI

A main effect regarding DSM-5 subtype ($F(2, 298) = 11.366$, $p < .001$) and a DSM-5 subtype × season interaction effect were found ($F(2, 298) = 6.119$, $p = .014$), whereas no main effect of season ($F(1, 298) = 6.232$, $p = .002$) was found on a (corrected) alpha-level of $\alpha = .00625$.

2. One-way ANOVAs to examine DSM-5 subtype effects within the seasons

The lowest BMI values of ANR were observed in the warm season, with a large difference of ANR from other AN subtypes ($F(2, 123) = 12.930$, $p < .001$), whereas in the cold season, AN subtypes did not vary regarding their BMI at admission ($F(2, 175) = 0.917$, $p = .402$).

3. Post-hoc t-tests to examine seasonal differences in BMI separate for DSM-5 subtypes

In the table, BMI values for the separate subtypes and seasons are reported. The effect of season does not reach significance when using BMI instead of BMI-SDS.

DSM-5 anorexia nervosa subtype	Cold season			Warm season					
	N	*M*	*SD*	*N*	*M*	*SD*	*t*	*df*	*p*
Restricting type	145	15.19	1.33	104	14.90	1.45	1.616	247	.107
Binge-eating purging type	7	15.93	1.09	6	17.35	1.40	-2.056	11	.064
Subclinical	26	15.23	1.92	16	16.51	2.32	-1.935	40	.060
