# Supplementary material for: Seasonal variation of BMI at admission in German adolescents with anorexia nervosa
Source: PLoS One. 2018 Sep 11;13(9):e0203844. doi: 10.1371/journal.pone.0203844 (PMC6133390; doi:10.1371/journal.pone.0203844)
Supplement: S2 File — List of all involved ethics committees. (PDF) [file pone.0203844.s002.pdf]

S2 File. Ethics statement. List of all involved ethics committees.

#	Study center	Ethics committee	Reference number
1	Essen	ethics committee of the medical faculty of the University Essen	12-5169-BO
2	Aachen	ethics committee of the medical faculty of the RWTH Aachen	EK028/12
3	Mainz	ethics committee of the local medical advisory board Rheinland-Pfalz	837.130.16 (10449)
4	Marburg	ethics committee of the medical faculty of the Philipps-University Marburg	86/14
5	Würzburg	ethics committee of the medical faculty of the Julius-Maximilians-University Würzburg	180/13_z
6	Dresden	ethics committee of the TU Dresden	EK 342092014
7	Freiburg	ethics committee of the Albert-Ludwigs-University Freiburg	132/14
8	Viersen	ethics committee of the local medical advisory board Nordrhein	2014183
9	Homburg	ethics committee of the local medical advisory board Saarbrücken	220/13
10	Berlin	ethics committee (board nr. 2) of the Charité Berlin	EA2/003/14
11	Heidelberg	ethics committee of the medical faculty Heidelberg	S-244/2015
12	Hamm	ethics committee of the medical faculty of the Ruhr-Universität Bochum	5082-14
13	Tübingen	ethics committee of the medical faculty of the Eberhard-Karls-Universität Tübingen	343/2014BO1
14	Ulm	ethics committee of the university of Ulm	170/07
15	Bonn	joint vote with center in Viersen	
16	Münster	ethics committee of the medical advisory board Westfalen-Lippe and the Westfälische Wilhelms-Universität	2016-384-b-S
